# Mortality and cause of death in hip fracture patients aged 65 or older - a population-based study

**DOI:** 10.1186/1471-2474-12-105

**Published:** 2011-05-20

**Authors:** Jorma Panula, Harri Pihlajamäki, Ville M Mattila, Pekka Jaatinen, Tero Vahlberg, Pertti Aarnio, Sirkka-Liisa Kivelä

**Affiliations:** 1 Department of Surgery, City Hospital of Pori, Pori, Finland; 2 Centre for Military Medicine, Helsinki, Finland; 3 Division of Orthopaedics and Traumatology, Department of Trauma, Musculoskeletal Surgery and Rehabilitation, Tampere University Hospital, Tampere, Finland; 4 Rauma Health Office, Rauma, Finland; 5 Department of Biostatistics, University of Turku, Turku, Finland; 6 Department of Surgery, Satakunta Central Hospital, Pori, Finland; 7 Department of Surgery, University of Turku, Turku, Finland; 8 Department of Family Medicine, University of Turku, Turku, Finland; 9 Department of Family Medicine, Turku University Central Hospital, Turku, Finland; 10 Department of Family Medicine, Satakunta Central Hospital, Pori, Finland

## Abstract

**Background:**

The high mortality of hip fracture patients is well documented, but sex- and cause-specific mortality after hip fracture has not been extensively studied. The purpose of the present study was to evaluate mortality and cause of death in patients after hip fracture surgery and to compare their mortality and cause of death to those in the general population.

**Methods:**

Records of 428 consecutive hip fracture patients were collected on a population-basis and data on the general population comprising all Finns 65 years of age or older were collected on a cohort-basis. Cause of death was classified as follows: malignant neoplasms, dementia, circulatory disease, respiratory disease, digestive system disease, and other.

**Results:**

Mean follow-up was 3.7 years (range 0-9 years). Overall 1-year postoperative mortality was 27.3% and mortality after hip fracture at the end of the follow-up was 79.0%. During the follow-up, age-adjusted mortality after hip fracture surgery was higher in men than in women with hazard ratio (HR) 1.55 and 95% confidence interval (95% CI) 1.21-2.00. Among hip surgery patients, the most common causes of death were circulatory diseases, followed by dementia and Alzheimer's disease. After hip fracture, men were more likely than women to die from respiratory disease, malignant neoplasm, and circulatory disease. During the follow-up, all-cause age- and sex-standardized mortality after hip fracture was 3-fold higher than that of the general population and included every cause-of-death category.

**Conclusion:**

During the study period, the risk of mortality in hip fracture patients was 3-fold higher than that in the general population and included every major cause of death.

## Background

Hip fracture is the most serious consequence of falling in older people with osteoporosis; 87% to 96% of hip fracture patients are 65 years of age or older [1,2]. Hip fractures are associated with increased mortality rates; the magnitude of the increased mortality and the length of its duration, however, are unclear. One study stated that survival declines soon after hip fracture, but thereafter parallels the expected survival of the general population [3]. A recent systematic epidemiologic review, however, showed that patients are at increased risk for premature death for many years after hip fracture [4].

Excess mortality after hip fracture may be linked to complications following the fracture, such as pulmonary embolism [5], infections [2,6], and heart failure [2,6]. Factors associated with the risk of falling and sustaining osteoporotic fractures may also be responsible for the excess mortality [1,7]. Excess mortality after fracture may be due to the individual characteristics of the person sustaining the hip fracture [8]; e.g., low-bone density is associated with increased non-trauma mortality, even without fractures. Despite numerous mortality studies, further analysis of mortality and cause of death is important to identify the risk factors for death following trauma and to anticipate complications [9].

Although several studies report excess mortality in hip fracture patients compared to controls [1] the issue remains under-recognized in many countries. Furthermore, sex- and cause-specific mortality after hip fracture has not been extensively investigated [6]. The aims of the present population-based study were to evaluate patterns of death by sex and hip fracture type and to evaluate mortality after hip fracture compared to the general population with a specific focus on the cause of death.

## Methods

The data analyzed in the present study were extracted from the records of hip fracture patients collected on a population-basis in the province of Satakunta in the western coastal region of Finland and from statistics on the general population collected on a cohort-basis of all persons 65 of age or older living in Finland. The referral area for hip fracture patients was the Satakunta Hospital District, which has a population of 235,580 (December 31, 1999). The number of residents 65 years of age or older in this district totalled 39,910 at the time of the study (16.9% of the population). Population figures for the hospital district and the entire country on December 31 in 1998 and in 1999 were obtained from the Official Statistics of Finland [10].

### Patients

The Finnish Hospital Discharge Register was used to retrieve data on people 65 years of age or older living in the Satakunta area who underwent hip fracture surgery during the 2-year period between January 1, 1999, and December 31, 2000. Of these, all patients who resided in the district during the study period were included in the study, irrespective of the operating hospital location. Patients who underwent surgery in the study area but did not reside there were excluded. The total number of eligible patients was 461. Of these patients, valid information could be collected for 428 patients. Thirty-three patients (10 men, 23 women) were excluded from the study due to missing death information. Of the 428 patients included in the present study, fracture-type data were missing in 3 men and 18 women. Data regarding preoperative comorbidities and operation type were retrospectively collected from the original patient records. Hip fractures were classified as cervical or trochanteric based on examination of the original radiographs.

### General Population

The general population comprised people who resided in Finland between January 1, 1999, and December 31, 2000, that were 65 of age or older during this period. Population data were divided into three age categories (65-74, 75-84, and ≥85 years).

### Deaths

Data on deaths and cause of death were retrieved from the Official Cause of Death Statistics of Finland [10]. Deaths in Finland must be reported immediately either to a physician or to the police. A death certificate is issued by a physician and delivered to the Provincial State Office, where it is checked and forwarded to Statistics Finland. The verification is made by a forensic pathologist or a specifically trained provincial physician. Death certificates are used at Statistics Finland to compile cause-of-death statistics [10].

Cause-of-death statistics are compiled from data obtained from death certificates, which are supplemented with data from the population information system of the Population Register Centre. These statistics cover persons who have died in Finland or abroad during the calendar year and who at the time of death were domiciled in Finland. Cause-of-death statistics contain data on deaths by cause of death, age, sex, marital status, and other demographic variables and data on the circumstances of the death [10]. The Finnish Official Cause-of-Death Statistics are in practice 100% complete, as each death, its certificate, and the corresponding personal information in this computerized population register is crosschecked [10].

### Mortality

Mortality of the hip fracture patients was assessed at the end of the follow-up, on December 31, 2007. Cause of death was classified according to the International Statistical Classification of Diseases and Related Health Problems (ICD10) [11] as follows: malignant neoplasms (ICD10 codes C00-C97); dementia (including Alzheimer's disease, ICD10 codes F01, F03, G30, R54); circulatory system disease (ICD10 codes I00-I42.5, I42.7-I99 [cerebrovascular disease included]); respiratory system disease (ICD10 codes J00-J64, J66-J99); digestive system disease, excluding alcohol-related disease (ICD10 codes K00-K93, excluding K70, K86.0, K86.01, K86.08); and other (ICD10 codes not mentioned above). Preoperative comorbidities were similarly classified.

Mortality of the general population was assessed on December 31 of each year from 1999 to 2007. For comparison with the general population, hip fracture patients were divided into three age categories (65-74, 75-84, and ≥85 years) and their annual mortality by sex was assessed from 1999 to 2007 to calculate age- and sex-standardized mortality. The person years (PY) were calculated by sex, age category, and calendar year.

The study protocol was approved by the Ethics Committee of the Satakunta Hospital District.

### Statistical analysis

Sex differences of continuous variables were compared using a two-sample *t*-test. A chi-square test was used to analyze the differences in baseline characteristics between men and women. The age-adjusted differences in mortality between sexes were analyzed with Cox's proportional hazards model. The age- and sex-adjusted differences in mortality between hip fracture types (n = 407) were analyzed with Cox's proportional hazards model. The survival time was counted in calendar days and expressed as person years from the day of the operation to the day of death or to the end of the follow-up. Results are presented using hazard ratios (HR) with 95% confidence intervals (95% CI). Annual age- and sex-standardized mortality of hip fracture patients was calculated for three age categories (65-74, 75-84, and ≥85 years) with a direct method based on the general population in Finland. A p-value of less than 0.05 was considered statistically significant. Statistical analyses were performed using the SAS System for Windows, version 9.1 (SAS Institute Inc, Cary, NC).

## Results

### Patient characteristics

Patient characteristics are shown in Table 1. A total of 428 hip fracture patients that were eligible for this study were operated on between January 1, 1999 and December 31, 2000, and the follow-up lasted until December 31, 2007. The mean follow-up period was 3.7 years (range 0-9 years). Most patients were women (n = 325, 75.9%), and they were older than the men (mean ages 82.7 [women], 79.0 [men] years; p < 0.001). Distributions of fracture and operation types were similar between sexes. The majority of fractures were cervical, and the numbers of prostheses and internal fixations were equivalent between groups. The majority of the patients (75%) died in a hospital, health center, or other care facility. Home or another dwelling was the place of death for 7 men (8.1%) and 7 women (2.8%; p = 0.031). The 21 patients (3 men and 18 women) with missing fracture-type data were older than the patients with identified fracture-type data (84.8 vs. 81.6 years, p = 0.041).

**Table 1:** Patient Characteristics (n = 428)

	Men	Women		
	**n(%)**	**n(%)**	**p-value**	

	103 (24.1)	325 (75.9)		
Age in years; mean (SD)	79.0 (7.0)	82.7 (6.7)	< 0.001	
Type of fracture*			0.421	
Cervical fracture	67 (67.0)	192 (62.5)		
Trochanteric fracture	33 (33.0)	115 (37.5)		
Type of operation*				
Prosthesis	54 (52.4)	174 (53.7)	0.821	
ORIF	49 (47.6)	150 (46.3)		
Preoperative morbidity				
Circulatory disease	64 (62.1)	227 (69.9)	0.144	
Respiratory disease	18 (17.5)	25 (7.7)	0.004	
Malignancy	17 (16.5)	23 (7.1)	0.004	
Dementia, Alzheimer's disease	15 (14.6)	93 (28.6)	0.004	
Digestive disease	6 (5.8)	25 (7.7)	0.524	
Other	17 (16.5)	45 (13.9)	0.504	
Follow-up time†; mean (SD)	3.3 (3.1)	3.9 (3.0)	0.077	
Died during follow-up	86 (83.5)	252 (77.5)	0.196	
Underlying cause of death				
Circulatory disease	39 (37.9)	110 (33.9)	0.456	
Respiratory disease	13 (12.6)	31 (9.5)	0.369	
Malignancy	12 (11.7)	19 (5.9)	0.048	
Dementia, Alzheimer's disease	10 (9.7)	43 (13.2)	0.344	
Digestive disease	2 (1.9)	14 (4.3)	0.270	
Other	10 (9.7)	35 (10.8)	0.760	
Place of death				
Hospital, health centre, other care facility	65 (75.6)	190 (75.4)	0.973	
Home, dwelling	7 (8.1)	7 (2.8)	0.031	
Other (e.g., old people's home)	14 (16.3)	54 (21.4)	0.304	
Unknown	0 (0)	1 (0.4)	0.559	

SD = standard deviation

ORIF = open reduction and internal fixation

* Type of fracture unknown for 21 patients and type of operation unknown for 1 patient

† Follow-up years from operation to death or end of 2007

Overall mortality (cumulative number of deaths) was as follows: at 30 days after surgery, n = 45 (10.5%); at 6 months, n = 92 (21.5%); at 1 year, n = 117 (27.3%); at 3 years, n = 209 (48.8%); at 7 years, n = 315 (73.6%); and on December 31, 2007 (end of follow-up), n = 338 (79.0%). Circulatory system disease was the most common cause of death (n = 149, 44.1%), followed by dementia and Alzheimer's disease (n = 53, 15.7%), respiratory system disease (n = 44, 13.0%), malignant neoplasms (n = 31, 9.2%), digestive system disease (n = 16, 4.7%), and other (n = 45, 13.3%).

### Sex differences in mortality and cause of death in hip fracture patients (n = 428)

The risk of death in men compared with women was increased during the follow-up with HR 1.55 (95% CI 1.21-2.00, p < 0.001).

During the entire follow-up (0-9 years), some differences in age-adjusted cause-of-death patterns between sexes were noted: men were more likely than women to die from respiratory system disease (HR 2.17, 95% CI 1.11-4.24, p = 0.023), from malignant neoplasms (HR 2.15, 95% CI 1.02-4.54, p = 0.044), and from circulatory system disease (HR 1.71, 95% CI 1.18-2.49, p = 0.005). Age-adjusted analysis of cause of death showed no sex differences in death from dementia and Alzheimer's disease, digestive system disease, or other causes (data not shown).

### Fracture-type differences in mortality and cause of death in hip fracture patients (n = 407)

In age- and sex-adjusted analyses, no differences in mortality or cause of death were noted between fracture types (data not shown).

### Comparisons of mortality and cause of death between hip fracture patients and the general population

During the follow-up from January 1, 1999, to December 31, 2007, age- and sex-standardized mortality was approximately 3-fold higher in hip fracture patients than in the general population (Table 2). When assessed by cause of death, age- and sex-standardized mortality was 2.5 to 8.4-fold higher in patients compared to the general population in each cause-of-death category (Table 2).

**Table 2:** Standardized Annual Mortality and Causes of Death in Hip Fracture Patients and Population 1999-2007*

	Patients (1,593 PY)	Population (6,089,623 PY)	RR
	Standardized mortality (95% CI)	Mortality	
Annual average	172.96 (142.26-203.65)	52.98	3.26
Circulatory system disease	69.82 (50.90-88.74)	25.31	2.76
Malignant neoplasm	26.01 (12.52-39.51)	10.30	2.53
Respiratory system disease	22.95 (11.32-34.59)	4.22	5.44
Dementia, Alzheimer's disease	17.59 (10.91-24.26)	5.77	3.05
Digestive system disease	13.41 (3.50-23.32)	1.59	8.43
Other	23.17 (12.05-34.29)	5.79	4.00

* Counted per 1000 person years (PY) with 95% confidence intervals (95% CI) in patients RR = relative risk of death for hip fracture patients compared to the general population

## Discussion

The present population-based study showed that during a 9-year follow-up, the age- and sex-standardized all-cause mortality of hip fracture patients was 3-fold higher than that of the general population. The increased mortality was related to every cause-of-death category, i.e., malignant neoplasm; dementia; diseases of circulatory, respiratory, and digestive systems; and other. Our study area lies in the western coast of Finland; there are regional differences of mortality in favor of Western Finland compared to Eastern Finland [12]. This aspect enhances the mortality difference between the hip fracture patients and the general population of our study. Of hip fracture patients, men had significantly higher mortality than women during the follow-up. Men were more likely to die from respiratory system disease, malignant neoplasms, and circulatory system disease after hip fracture than women. Sex differences in cause of death after hip fracture have not been systematically studied [6]. To our knowledge, there are no studies of mortality after hip fracture surgery with follow-up periods of several years in which cause of death was analyzed in association with sex.

In the present study, the increased age-adjusted mortality in men compared to women was obvious immediately after the hip fracture operation and persisted until the end of the follow-up, which is consistent with previous studies [6,13,14]. The reasons for the sex differences are unclear, although men tend to be sicker and frailer than women at the time of fracture [15]. Greater impairments in activities of daily living, mobility, and walking speed have been observed in male hip fracture patients compared to women. This may indicate a greater loss of physiologic reserve after hip fracture in men than in women and, hence, a greater risk of death [14].

This study revealed differences between sexes in cause of death after hip fracture. Men in our study were more likely than women to die from respiratory diseases. At baseline, respiratory diseases were more common in men than in women. Patient's history of smoking was not systematically recorded in the original patient records, and no data on smoking were collected. In Finland, however, smoking is more common in elderly men than in elderly women [16]. One explanation for the excess male mortality is the assumption that men are more prone to exacerbation of respiratory problems after hip fracture surgery (decreased secretion in the airways, impaired activities of daily living, and subsequent chest complications). In addition, smoking may be related to another finding of our study. Malignant neoplasms were more likely the cause of death for men than for women. A recent cancer survey in Europe concluded that differences in cancer mortality between sexes and European countries may be explained by smoking habits [17].

Some previous studies indicate that patients with heart disease may be more likely to fall and thus sustain a hip fracture as a consequence of impaired circulation, but impaired circulation may also increase the likelihood of dying after having sustained a fracture [1,15]. Similarly, patients who are immobilized and those with osteoporosis following a stroke may not only have an increased risk of falls and fractures, but also an increased risk of dying following complications related to neurovascular disease [18]. The reason for the greater risk of death from circulatory disease in men after hip fracture cannot be addressed on the basis of the present study. In Finland, coronary heart diseases were previously very common in middle-aged men, but the occurrence has decreased over the past 10 to 20 years. In women, these diseases usually occur at an older age than in men [19]. Hence, circulatory system disease might be more severe and more long-term in older men than in older women.

Findings from analyses by fracture type showed no differences between cervical and trochanteric fracture patients in mortality or cause of death. In contrast, a Greek study of 499 hip fracture patients with trochanteric fracture predominance (67%) reported higher mortality after trochanteric hip fracture than after cervical hip fracture at 5 and 10 years after the incident [20]. Their conclusion was that the type of hip fracture was an independent predictor of long-term mortality in hip fracture patients. A Danish study of 2674 hip fracture patients with cervical hip fracture predominance (64%), however, reported that the mortality rates between cervical and pertrochanteric hip fracture patients were not significantly different during a mean follow-up of 2.6 years [21].

Rehabilitation of older patients falls under the purview of primary health care; geriatric rehabilitation is poorly established in Finland. After surgery, hip fracture patients are usually referred to local hospital wards for primary care, where the treatment is conservative with no special knowledge of modern geriatrics. A Finnish randomized, controlled intervention study of patients 65 years of age or older sustaining hip fracture showed that active rehabilitation performed by a geriatric team shortened the total hospital stay after a hip fracture operation and enhanced the recovery of daily activities [22]. The lack of a geriatric rehabilitation center in the Satakunta area may be one explanation for the high mortality of hip fracture patients of our report.

The accuracy of registering severe injuries like hip fractures is generally good in Finland. The completeness and accuracy of data from the Finnish Health Care Register and the Cause-of-Death Register are suitable for assessing hip fracture treatment [23]. Complete follow-ups of both fracture patients and population are strengths of our study. It may also be assumed that surgical practices, anaesthetics, and postoperative treatment remained unchanged during the 2-year catchment period of fracture patients. A control cohort with corresponding age included the whole Finnish population and its mortality was registered comprehensively. To our knowledge, such a comparison has not previously been made in the English literature.

Our study also has several limitations. Persons with medical and functional deficits are more likely to sustain hip fracture than healthy people; moreover, they have an increased mortality risk even without hip fracture. This might result in an overestimated risk of death in hip fracture patients compared to the general population. The other problem related to comorbidities might be the difficulty in identifying the "ultimate" cause of death. Data on patient comorbidities were retrospectively collected from the original patient records, which are usually based on the information received from the previous hospital records, referral documents, and patient and/or proxy interviews. They may not always be reliable or fully comprehensive. We had no information on the frequency of autopsies in cases and controls or other possible differences in determination of the cause of death. In general, autopsies are seldom performed in older people. Furthermore, we did not have information on postoperative complications or functional recovery. According to Vestergaard et al. [1], the major causes of the excess mortality after fracture were complications related to the fracture event. Because there are regional differences of mortality in Finland, patterns of death of hip fracture patients and the general population in Satakunta should be analyzed in future studies.

Our study analyzed excess mortality, i.e., deaths due to hip fracture that might be prevented, from the scope of cause of death. To reduce mortality after hip fracture, optimal treatment of all major comorbidities should be emphasized. One measure toward achieving this goal might be improved treatment after discharge from hospital as primary health care becomes responsible for the treatment. Specialist medical assessment and management of older people with hip fracture before and after surgery have been recommended [2]. Interventions such as nutritional supplementation and dietetic assessment, comprehensive multidisciplinary intervention programs, and in-hospital programs might improve outcomes, including mortality after hip fracture [24,25]. Furthermore, enrolling specially educated personnel in hospitals treating fractures might improve the secondary prevention of fractures [26]. Most of these measures, however, are hospital-focused and long-term cooperation between primary health care and specialist health care needs to be enhanced to improve survival after hip fracture.

## Conclusion

The present study showed that during the follow-up up to 9 years, the age- and sex-standardized all-cause mortality of hip fracture patients was 3-fold higher than that of the general population. A similar trend was observed for each cause-of-death category.

## Competing interests

The authors declare that they have no competing interests.

## Authors' contributions

Each author has made substantive intellectual contributions to this study:

JP: writing manuscript, acquisition and interpretation of data, statistical analysis; HP: design of study, manuscript revision; VM: statistical analysis, interpretation of data, manuscript revision; TV: statistical analysis; PJ, PA, SLK: conception of data, manuscript revision. All authors read and approved the final manuscript.

## Pre-publication history

The pre-publication history for this paper can be accessed here:

http://www.biomedcentral.com/1471-2474/12/105/prepub
